# A Recognition Method of Truck Drivers’ Braking Patterns Based on FCM-LDA2vec

**DOI:** 10.3390/ijerph192315959

**Published:** 2022-11-30

**Authors:** Jianfeng Xi, Yunhe Zhao, Zhiqiang Li, Yizhou Jiang, Wenwen Feng, Tongqiang Ding

**Affiliations:** 1 College of Transportation, Jilin University, Changchun 130022, China; 2 China Academy of Transportation Sciences, Beijing 100029, China

**Keywords:** truck operation data, braking behavior, braking pattern, FCM and LDA2vec

## Abstract

Taking truck drivers’ braking patterns as the research objects, this study used a large amount of truck running data. A recognition method of truck drivers’ braking patterns was proposed to determine the distribution of braking patterns during the operation of trucks. First, the segmented data of braking behaviors were collected in order to extract 25 characteristic parameters. Additionally, seven main correlation factors were obtained by dimensionality reduction. The FCM clustering algorithm and CH scores were used to identify nine categories of truck drivers’ braking behaviors. Then the LDA2vec model was used to identify the distribution of different braking behavior words in braking patterns, and three categories of truck drivers’ braking patterns were identified. The test results showed that the accuracy of the truck drivers’ braking pattern recognition model based on LDA2vec was higher than 85%, and braking patterns of drivers in the daily operation process could be mined from vehicle operation data. Furthermore, through the monitoring and pre-warning of the braking patterns and targeted training of drivers, traffic accidents could be avoided. At the same time, this paper’s results can be used to protect human life and health and reduce environmental pollution caused by traffic congestion or traffic accidents.

## 1. Introduction

Since trucks have the characteristics of long vehicle length, large load capacity, and large volume, the severity of road traffic accidents related to trucks is often relatively large, so the study of truck accidents is crucial. Studies have shown that among the causes of truck accidents, truck drivers’ braking, over-speeding, and fatigued driving account for the highest proportions [1,2]. Hu Liwei et al. studied the complex relationship between truck operation risk factors, including truck drivers’ fatigued driving behavior and dynamic reaction judgment ability and other drivers’ own risk factors, and the strength of dynamic response judgment ability can be reflected in braking behavior [3]. Based on the human factor analysis and classification system, Zheng Shibo and other scholars proposed an analysis model for the causes of truck traffic accidents, indicating that driver factors are the main causes of truck traffic accidents [4]. That is to say, analysis of truck drivers’ behaviors in the driving process is vital in studying traffic safety. Regarding the analysis of driving behaviors, it is possible to do post-event analysis based on traffic accident data or accident cases. However, are there inherent driving behavior characteristics or hidden risk points in the truck-operating data before the accidents? The answer is yes. For example, truck drivers brake to avoid dangers or even accidents in the process of driving. The braking behavior may be such a hidden risk point, that is to say, the data or laws related to the braking behavior are hidden in the daily operation of trucks.

The research on braking behaviors mainly focuses on analyzing the braking process of vehicles under specific traffic scenarios. It is roughly divided into the research on the driving conflict between the motor and non-motor vehicles [5,6], drivers’ braking behaviors under distractions [7], normal braking process [8], and behavioral analysis of drivers’ braking during emergency braking [9,10]. There are relatively few targeted studies on the braking behaviors of trucks.

With the collection of vehicle operation data becoming more and more popular, the analysis of vehicle operation data has gradually become a hot spot in traffic safety research. On the one hand, the GPS track data of trucks are used to study the driving behaviors and operating states of truck drivers [11,12]. On the other hand, based on the operating parameters generated during the operation of trucks, such as the speed, acceleration, angular velocity, brake pedal level, accelerator opening, and driving time, these data are comprehensively analyzed to mine truck-operating characteristics and drivers’ behavior characteristics [13,14].

From the perspective of the truck operation process, similarities and differences in the sequence or frequency of emergency braking, normal braking, and moderate braking in a segment of braking data reflect the similarities and differences in the distribution of truck drivers’ braking behaviors. This further indicates that different truck drivers have different degrees of traffic safety risk in the process of driving. Therefore, in order to screen the driving risk degree of truck drivers before an accident, it is necessary to extract the potential characteristics and rules of truck drivers’ braking behaviors in the daily operation process from the long-term operation data of trucks.

This study separates the braking behavior segment data from the truck operation data, and proposes a framework model to identify the truck driver’s braking pattern. In this way, the distribution characteristics of truck drivers’ braking behaviors and the distribution law of braking patterns in the daily operation process are extracted. According to the research results, a brake behavior detection and early warning model for truck drivers can be developed, or truck driver education and awareness can be increased to reduce the occurrence of truck accidents.

## 2. Data

### 2.1. Data Collection

The basic data of the natural driving of trucks in this study came from the vehicle monitoring platform of one logistics company, which contains multi-dimensional data items (see Table 1 for details).

The time interval of data collection was 1 s. The natural driving data of 20 truck drivers were randomly selected, and the data sample size was about 1.82 million. Abnormal, missing, and erroneous data within the basic data were cleaned and processed, and a series of denoising processes were performed on the data by wavelet deposition, threshold processing, and reconstruction [15]. Next, a total of 870 segments of the braking behavior were extracted from the processed dataset. The braking behavior segment data of each truck driver was combined as a segment of braking data, with a total of 20 segments of braking data obtained.

### 2.2. Extraction and Dimensionality Reduction of Characteristic Parameters

The characteristics parameters were extracted from each braking behavior segmented data to obtain 25 characteristic parameters of each braking behavior segment, thus reflecting the horizontal and vertical changes in truck drivers’ braking behaviors (see Table 2 for specific characteristic parameter items).

There was a large difference between the values of different parameter items in the truck-operating data. The min–max normalization method was used to normalize the characteristic parameters of these 25 braking behavior segments of truck drivers to avoid the small data being weakened by the big data due to the great disparity of values.

These 25 feature parameters belong to high-dimensional data. The dimensionality of the 25 characteristic parameters were reduced to facilitate the subsequent data analysis of the truck drivers’ braking behaviors and patterns. Factor analysis (FA) was used to reduce the dimensionality of parameters and excavate the internal correlation and potential common factors of parameter variables [16]. After factor analysis, seven main correlation factors were extracted from the original 25 characteristic parameters (see Table 3 for the score coefficients of each main correlation factor).

The process of factor analysis was:

Step 1: Suitability discrimination for factor analysis. If the value of KMO (Kaiser-Meyer-Olkin) was greater than 0.6, the braking behavior data parameters of truck drivers were considered suitable for factor analysis.

Step 2: Factor and parameter correspondence discrimination. If the correspondence between the factor and the parameter was seriously inconsistent with the expectations, the parameter item could be considered for deletion.

Step 3: Determination of the main correlation factor. In the second step, unreasonable parameters were removed and the factor confirmed, and once the remaining parameters corresponded well, the main correlator could be determined.

Factors F_1_, F_3_, and F_6_ mainly express the changes in lateral acceleration and angular velocity in the braking process of truck drivers, reflecting the severity of lateral turnings. Factors F_2_ and F_4_ show the changes in the longitudinal speed and acceleration in the braking process of truck drivers, indicating the severity of longitudinal speed changes. Factors F_5_ and F_7_ mainly express the relative changes between the truck driver’s braking process and target distance and the braking time, presenting the risk degrees of braking behaviors.

## 3. Methods

### 3.1. Frame Model

Two issues need to be considered to extract the braking pattern/behavior distribution in the braking pattern from the data. One is that in order to identify braking patterns from truck braking data, the types of braking behavior in each piece of braking data should be known. The data based on the cluster analysis of the truck drivers’ braking behavior parameters are high-dimensional, with a correlation between parameters. The fuzzy c-means (FCM) clustering algorithm can be used to address these problems to obtain the optimal clustering results. Therefore, the FCM clustering algorithm was used to determine the categories of braking behaviors in the truck braking data.

Another issue is that after determining the types of truck drivers’ braking behaviors, the distribution law of the truck driver’s braking patterns is determined by exploring the method of identifying the braking patterns from the braking data. Currently, there are two typical topic models, namely probabilistic latent semantic analysis (PLSA) [17] and latent Dirichlet allocation (LDA) [18]. In the process of training parameters, the LDA model does not become more complicated with the increased data set, and the calculation is relatively simple. Therefore, the LDA model was used to analyze the braking patterns of truck drivers.

However, if only the distribution of various braking behaviors in the braking patterns is studied, and the various braking behaviors are regarded as conditionally independent of each other, it is possible to ignore the sequence of braking behaviors, the sequence position of the same braking behavior, etc. Therefore, it is necessary to establish a relationship among braking data, braking behaviors, and braking patterns. Additionally, the braking behavior word sequence should be included to form the vectors of braking behavior words, which are trained by Word2Vec [19]. The advantages of Word2Vec and LDA were combined to identify truck drivers’ braking patterns.

**Figure 1:**
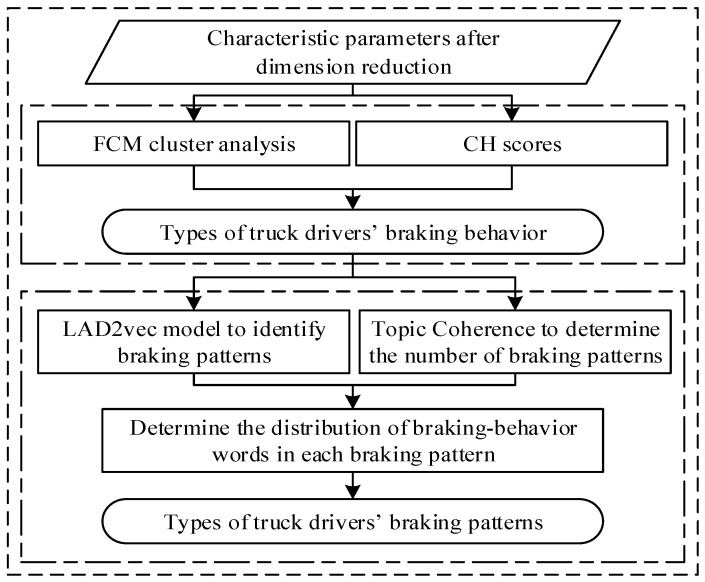
Framework of the truck drivers’ braking pattern recognition model.

In summary, a framework model for recognition of truck drivers’ braking patterns was proposed based on the FCM algorithm and LDA2vec model (see Figure 1). Additionally, the FCM clustering algorithm was used to analyze the types of braking behaviors from the related fragmented data, and the LDA2vec model was used to mine the distribution of braking patterns in the braking data and the distribution rules of braking behaviors in the braking patterns.

### 3.2. Braking Behavior Clustering Method

The FCM algorithm [20] was used in the cluster analysis of truck drivers’ braking behaviors. It was to give each data point of the truck drivers’ braking behaviors a membership function belonging to each category, and the membership values of the truck drivers’ braking behavior data were compared for classification.

In the cluster analysis process of the FCM algorithm, some categories need to be determined first. Calinski-Harabasz (CH) scores can be calculated by using the function in Scikit-learn, which is more efficient [21]. Therefore, the CH score index was selected to evaluate the clustering results of truck drivers’ braking behaviors. The ideal clustering effect is as follows: smaller covariances are suitable for the data within a category, while larger covariances fit the data between categories. Therefore, the larger the CH score, the better the clustering effect.

The cluster analysis process of braking behaviors is detailed in Algorithm 1.
**Algorithm 1**: Cluster AlgorithmsStep 1: CH scores determine the number of categoriesStep 2: FCM algorithm for cluster analysis   Input: Truck braking behavior data $X=\left\{x_{1},x_{2},\cdots ,x_{N}\right\}$, number of categories *K*, and threshold terminating iterations ε.   Initialization: Take the random value of [0, 1] to initialize membership degree matrix *U*_0_; assume that the initial value of the number of iterations is *h* = 1.   Iterations: Solve the cluster center based on Equation (1).        Solve the new membership degree based on Equation (2).        Solve the objective function based on Equation (3).        *h* = *h* + 1.   Conditions for terminating iterations: $\left|J^{h+1}-J^{h}\right|\leq \varepsilon$, where ε is usually 0.0000001.   Output: Cluster results

The equations are as follows.

(1) $$c_{v}=\frac{\sum_{n=1}^{N}u_{nv}^{K}\times x_{n}}{\sum_{n=1}^{N}u_{nv}^{K}}$$

(2) $$u_{nv}=\frac{1}{\sum_{i=1}^{V}{\left(\frac{\left\|x_{n}-c_{v}\right\|}{\left\|x_{n}-c_{i}\right\|}\right)}^{\frac{2}{K-1}}}$$

(3) $$J_{K}=\overset{N}{\underset{n=1}{\sum }}\overset{V}{\underset{v=1}{\sum }}u_{nv}^{K}\|x_{n}-c_{v}\|{}^{2},1\leq K<\infty$$

where *N* is the number of braking behavior data points; $x_{n}$ is the value of braking behavior data points; *V* is the number of cluster centers; $c_{v}$ is the value of the cluster center; *K* is the number of cluster categories; $u_{nv}^{K}$ is the membership degree of $x_{n}$ to $c_{v}$ when the braking behavior data is divided into *K* categories. $J_{K}$ is the sum of squared errors from the sample to various center points.

CH scores are calculated as

(4) $$CH\left(K\right)=\frac{tr\left(Q_{K}\right)}{tr\left(R_{K}\right)}\times \frac{N-K}{K-1}$$

where *N* is the number of the data; *K* is the number of cluster categories; $Q_{K}$ is the discrete matrix between categories (see Equation (5)); $R_{K}$ is the discrete matrix in categories (see Equation (6)).

(5) $$Q_{K}=\overset{K}{\underset{k=1}{\sum }}n_{k}\left(c_{k}-c_{X}\right){\left(c_{k}-c_{X}\right)}^{T}$$

(6) $$R_{K}=\overset{K}{\underset{k=1}{\sum }}\underset{x\in C_{k}}{\sum }(x-c_{k}){\left(x-c_{k}\right)}^{T}$$

where $n_{k}$ is the number of data in category *k*; $c_{k}$ is the cluster center of category *k*; $c_{X}$ is the center of all data sets *X*; $x\in C_{k}$ indicates that the current point is in category *k*.

### 3.3. LDA2vec Model to Identify Braking Patterns

(1) Word2Vec was used to build a braking behavior dictionary.

**Figure 2:**
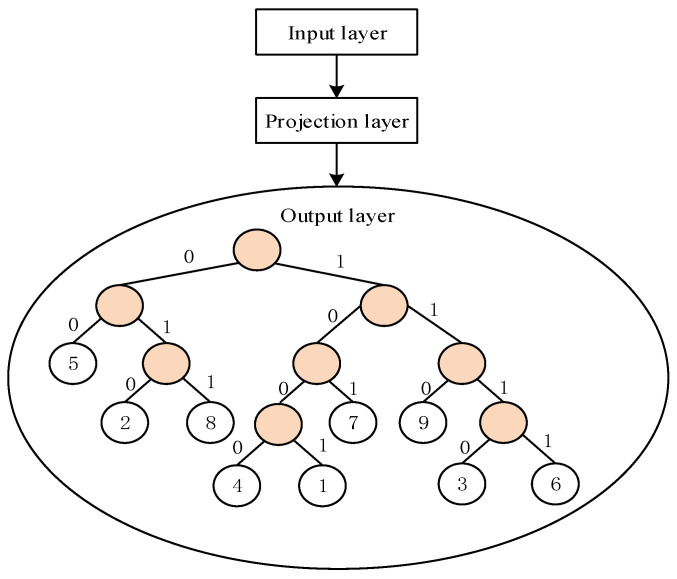
Structure of training truck drivers’ braking behavior words.

Word2Vec is a simple neural network with three layers including the input layer, projection layer, and output layer. The set of all braking behaviors constitutes a braking behavior dictionary, and each braking behavior is a word. The skip-gram model was used to train the word vectors for braking behaviors. Figure 2 shows its structure, and the meaning of each layer is as follows.

Input layer: The word vector of braking behavior words in the braking behavior dictionary.

Projection layer: In the skip-gram model [22], the projection layer is an identical projection process. That is, the word vectors of the braking behavior words in the input layer are projected to the new space.

Output layer: A Huffman tree [23] is output, and all classifications of braking behavior words should be in leaf nodes.

(2) LDA model to identify braking patterns

The LDA model contains the following aspects: Set $E=\left\{e_{1},e_{2},\cdots ,e_{S}\right\}$ contains the braking data of the truck drivers in segement *S*. Each segment of braking data *e* contains *A* braking behavior words. $O=\left\{o_{1},o_{2},\cdots ,o_{A}\right\}$, and each braking behavior word *o* comes from a dictionary containing *G* braking behavior words. Assuming that there are *T* types of the topics of truck drivers’ braking patterns, the braking pattern set is $Z=\left\{z_{1},z_{2},\cdots ,z_{T}\right\}$. The distribution of the above data sets is as follows.

① In all braking patterns, each segment of braking data *e* is subject to polynomial distribution ϑ. The construction of braking pattern *z* is based on the probability distribution of parameter ϑ, and polynomial distribution ϑ of the braking patterns of each segment of the braking data obeys the Dirichlet distribution. Its hyperparameter is α, that is, ϑ:Dirichlet(α). The mixing coefficient of each braking pattern in braking data *e* is obtained based on α.

② In the braking behavior dictionary, each braking pattern *z* obeys multinomial distribution. The probability distribution of braking behavior word *o* is constructed based on parameter ψ, and multinomial distribution ψ of *o* in the braking behavior dictionary obeys the Dirichlet distribution. Its hyperparameter is β, that is, ψ:Dirichlet(β). β is used to get the mixed coefficient of each braking behavior word in braking pattern *z*.

③ For each segment of braking data *e*, braking pattern *z* is obtained based on probability parameter ϑ, and braking behavior word *o* is obtained based on probability parameter ψ.

The LDA model applied to identify the truck drivers’ braking patterns outputs the probability distribution of the braking patterns of each segmented braking data in the braking data set. The steps for generating braking dataset *E* are as follows.

Step 1: Obtain the number of braking behavior words (*A*) in the braking data.

Step 2: Determine the number of braking patterns (*T*). The *T* value can be obtained through learning and training, and is constant.

Step 3: Determine the mixing coefficient of each braking pattern in braking data *e* by sampling the mixture ratio of each braking pattern. Its distribution is ϑ:Dirichlet(α).

Step 4: Determine the mixing coefficient of each braking behavior word in each braking pattern *z* by sampling the mixing ratios of each braking behavior word. Its distribution is ψ:Dirichlet(β).

Step 5: Generate $o_{A}$ of *A* braking behavior words in the braking data. Determine corresponding braking pattern $z_{A}$ of each $o_{A}$ by sampling; $z_{A}$ obeys multinomial distribution $z_{A}:Multinomial\left(\vartheta \right)$. Then determine braking behavior word $o_{A}$ by conditional probability $P(o_{A}|z_{A},\psi )$.

**Figure 3:**
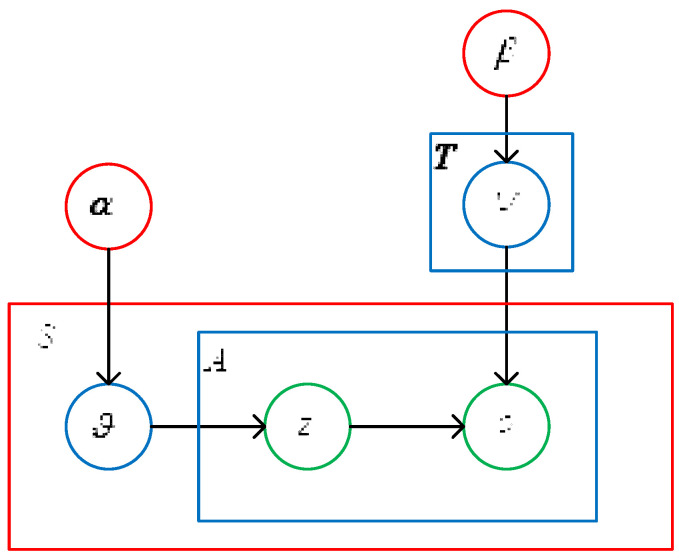
Implication of the LDA model.

Figure 3 shows the LDA model used for recognizing truck drivers’ braking patterns.

α and *β* are hyperparameters of the Dirichlet distribution. *S* is the number of segments of the truck driver’s braking data. *T* is the number of brake patterns. *ϑ* is polynomial distribution of brake patterns for each segment of brake data on all brake patterns, in this case Dirichlet distribution. ψ is the polynomial distribution that each braking mode obeys in the brake behavior dictionary, in this case the Dirichlet distribution. *A* is the number of brake behavior words; *z* is the brake mode; *o* is the word for braking behavior.

Recognizing truck drivers’ braking patterns based on the LDA model lies in solving hyperparameters α and β. Equation (7) shows the mixing coefficient of various braking patterns (ϑ), that of braking behavior words (ψ), braking pattern *z*, and the joint probability distribution of braking behavior word *o*.

(7) $$P(\vartheta ,z,\psi ,o|\alpha ,\beta )=P(\vartheta |\alpha )P(\psi |\beta )\overset{A}{\underset{i=1}{\prod }}P(z_{i}|\vartheta )P(o_{i}|z_{i},\psi )$$

where the Gibbs sampling algorithm [24] is used to sample the real truck braking data. Observable, implicit, and unknown variables are subjected to multivariate joint distribution. Parameters are obtained by the approximate solution to establish a model, thus clarifying the braking patterns of each segment of the truck braking data.

In the truck drivers’ braking pattern recognition based on the LDA model, the steps of the Gibbs sampling algorithm are as follows.

Step 1: Extract a braking behavior word from the truck braking dataset in a fixed order.

Step 2: Calculate the conditional probability that the extracted braking behavior words belong to a braking pattern with all the given remaining braking behavior words and braking patterns.

Step 3: Randomly select a braking pattern to replace that of the current braking behavior words.

Step 4: Repeat the above three steps until α and β converge to a fixed value.

During identification of the truck drivers’ braking patterns, the number should be determined. Topic coherence can evaluate the correlation between words [25]. The higher the topic consistency score, the more suitable the topic number corresponding to topic coherence as the number of truck drivers’ braking patterns (see Equation (8)).

(8) $$coherence\left(z,o^{z}\right)=\overset{T}{\underset{k=2}{\sum }}\overset{k-1}{\underset{i=1}{\sum }}log\frac{P_{2}\left(o_{k}^{z},o_{i}^{z}\right)+\delta }{P_{1}\left(o_{i}^{z}\right)}$$

where $o^{z}$ is the set of braking behavior words in braking pattern *z*; $P_{1}\left(o_{i}^{z}\right)$ is the frequency of braking behavior words; $P_{2}\left(o_{k}^{z},o_{i}^{z}\right)$ is the co-occurrence frequency of braking-behavior words $o_{i}^{z}$ and $o_{k}^{z}$; δ usually takes 1.

## 4. Results

### 4.1. Braking Behaviors Cluster

Multiply the 25-item initial parameter variable matrix of the 870 braking behavior segmented data and the coefficient matrix of main correlation factor scores to obtain the input parameter matrix of 7 items of cluster analysis, including F_1_, F_2_, F_3_, F_4_, F_5_, F_6_, and F_7_. The FCM algorithm was used to analyze the braking behaviors of truck drivers.

**Figure 4:**
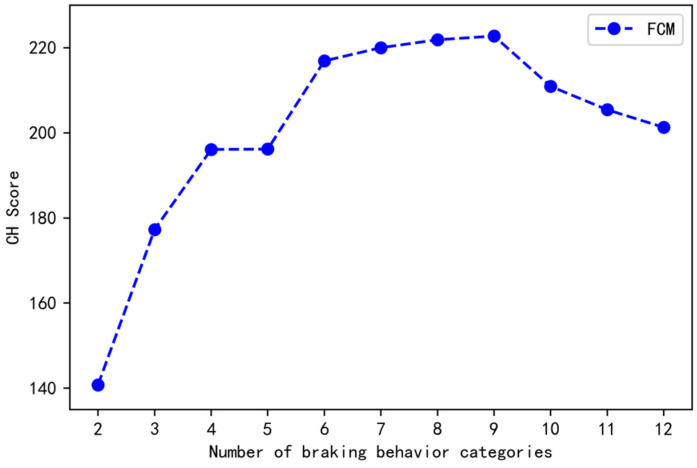
CH scores of truck drivers’ braking behavior cluster analysis.

Figure 4 shows the CH scores of the FCM algorithm from 2 to 12 categories, and the CH score with 9 categories is the maximum. Therefore, it is optimal to cluster the braking behavior of truck drivers into 9 categories.

**Figure 5:**
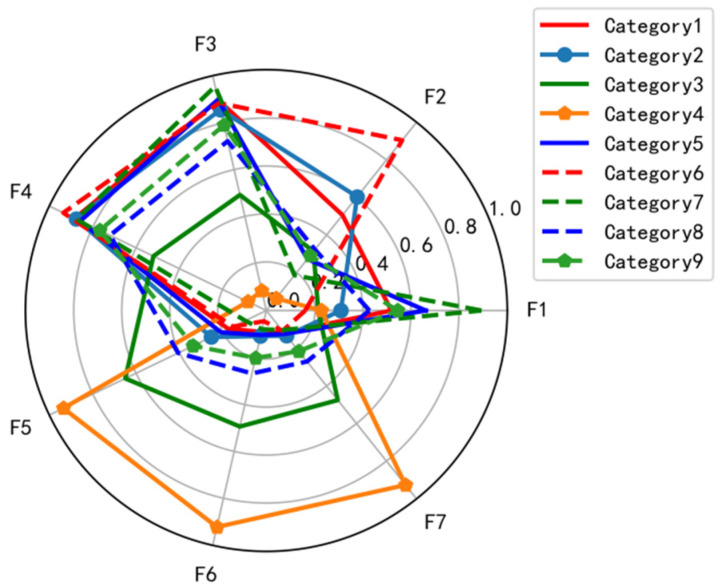
Cluster centers of nine cluster categories.

Figure 5 shows the radar chart of cluster center analysis in the nine categories. The boundaries for classifying the cluster centers between parameters are relatively clear, and the boundaries of the cluster center values between the categories are distinct. Thus, it is feasible to cluster the braking behaviors of truck drivers into nine categories.

F is the main correlation factor obtained after the above data processing factor analysis, which will be further explained in the article.

The nine categories correspond to nine types of braking behaviors of truck drivers, which can be defined as moderate straight braking, normal straight braking, emergent straight braking, moderate left-turn braking, normal left-turn braking, emergent left-turn braking, moderate right-turn braking, normal right-turn braking, and emergent right-turn braking.

### 4.2. Braking Pattern Recognition

Considering the insufficient sample size of braking behavior fragment data, five-fold cross-validation is used to identify the braking pattern. In each recognition training process, 16 segments of braking data are used as the training set, and 4 segments of braking data are used as the test set.

**Figure 6:**
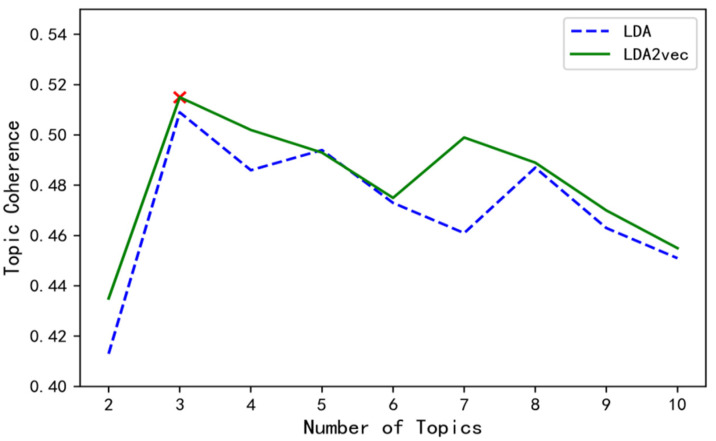
Topic coherence scores of the LDA and LDA2vec models.

The topic number of truck drivers’ braking patterns is determined using topic coherence. The higher the correlation between words within the same topic, the better the classification effect, so the higher the topic consistency score, the more suitable the number of topics corresponding to the topic consistency indicator as the number of categories in the truck driver’s braking mode. The LDA and LDA2vec models are used to identify the truck driver’s braking patterns (see Figure 6 for their respective topic coherence scores). In Figure 6, whether it is in the LDA model or the LDA2vec model, the topic coherence score is the maximum when the number of topics is three. Therefore, the three classes of the truck drivers’ braking patterns are selected.

**Figure 7:**
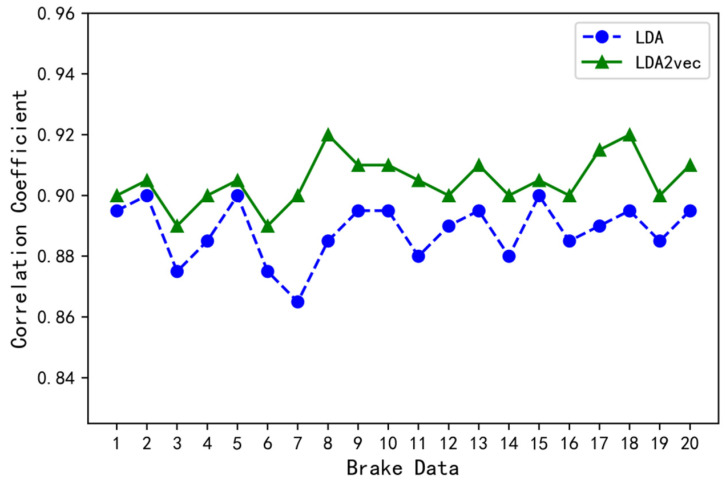
Correlation coefficients between the reconstructed and original braking data in the LDA and LDA2vec models.

Combined with the distribution of braking patterns and braking behavior words in the braking data, that is, the training results of the two models, the braking data are reconstructed. Correlation analysis is performed between the reconstructed and original braking data (see Figure 7 for correlation coefficients). The correlation coefficient of the training results based on the LDA2vec model was higher than that based on the LDA model, indicating that the distribution of the braking pattern and braking behavior words in the braking data in the LDA2vec model was more consistent with the distribution in the original data. The LDA2vec model has more advantages than the LDA model for identifying the truck drivers’ braking patterns.

**Figure 8:**
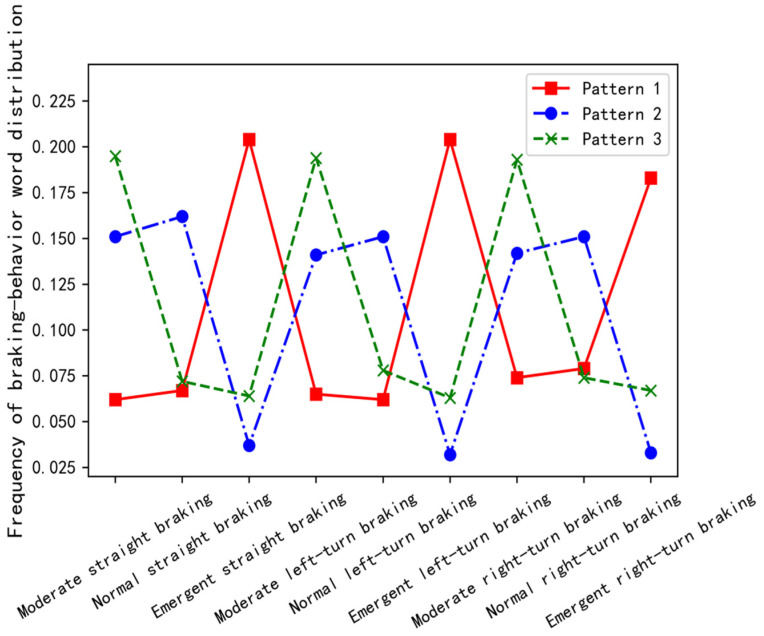
Distribution of different braking behavior words in three braking patterns.

Figure 8 shows the distribution of different braking behavior words in the three braking patterns based on the LDA2vec model. In braking pattern 1, emergent straight braking, emergent left-turn braking, and emergent right-turn braking, the frequency of the three brake behavior words was significantly higher, so it was judged to be impulsive braking. Similarly, in braking pattern 2, normal straight braking, normal left-turn braking, and normal right-turn braking, the frequency of the three brake behavior words was significantly higher, so it was judged to be smooth braking. In braking pattern 3, moderate straight braking, moderate left-turn braking, and moderate right-turn braking, the frequency of the three brake behavior words was significantly higher, so it was judged to be gentle braking.

### 4.3. Model Validation

The LDA and LDA2vec models are used to recognize the braking patterns, respectively (see Table 4 for the comparison results of recognition accuracy). Table 4 shows that the recognition accuracy for the three braking patterns of the LDA2vec model was generally higher than that of the LDA model, and both were above 85%. The LDA2vec model is more suitable and superior to the traditional LDA model for identification. The distribution of braking patterns can be better identified from the truck drivers’ braking data, which can determine the type of braking patterns in the truck brake data.

## 5. Conclusions

Based on massive truck operating data, this study proposed a method of truck drivers’ braking pattern recognition based on the FCM algorithm and the LDA2vec model to determine the distribution of braking patterns.

(1) The FCM algorithm and CH scores were used to define nine categories of truck drivers’ braking behaviors.

(2) Based on the clustering results of braking behaviors in the braking data, the LDA2vec model was used to determine the three braking pattern categories: impulse, smooth, and gentle. According to the distribution rules of different braking behavior words in braking patterns, three types of braking patterns were defined.

(3) The accuracy of the proposed truck drivers’ braking pattern recognition model was verified using the test set data. Both the LDA and LDA2vec models were suitable for identifying the truck drivers’ braking patterns, and the latter had more advantages.

The research ideas and methods described in this paper can be used to extract the truck operation characteristics and hidden risk information from the truck operation data and provide a reference for truck drivers’ traffic safety evaluation, early risk warning, safety education and training, etc., so as to improve the operation safety of truck drivers and reduce the safety risks of truck operation.

However, due to the limited extraction conditions of truck-operating data, this study does not eliminate a very small amount of abnormal data by playing back a huge amount of videos to compare the road traffic environment corresponding to braking behaviors. The braking behavior segmented data selected by the study may contain a small amount of natural deceleration of trucks. Therefore, it will slightly affect the accuracy and reliability of the cluster analysis and pattern recognition in the work.

In follow-up research, the number of surveyed drivers should be increased to ensure the integrity of the data; the road conditions and weather conditions should be considered; and real vehicle experiments should be used to collect data or synchronously analyze traffic video, which can not only explore the influence of road and traffic environmental factors on the braking behavior of truck drivers but also comprehensively analyze the purpose and intention of truck drivers’ braking behaviors.

## Figures and Tables

**Table 1:** Basic data of natural driving of trucks.

Data Item	Data Item
Vehicle ID	Longitudinal acceleration
License plate number	Target distance
Time	Relative target speed
System alarm level	Dangerous target ID
Braking force level	Left turn indicator status
Braking pedal status	Current position
Heading angle	Longitude
Speed	Latitude
Yaw angle	Number of satellites
Lateral acceleration	

**Table 2:** List of characteristic parameters of the truck drivers’ braking behavior segment.

Characteristic Parameter Item		Definition of Parameters	Characteristic Parameter Item		Definition of Parameters
Speed	Mean	v-mean	Longitudinal acceleration	Mean	az-mean
	Median	v-median		Median	az-median
	Maximum value	v-max		Maximum value	az-max
	Minimum value	v-min		Minimum value	az-min
	Variance	v-s^2^		Variance	az-s^2^
Lateral acceleration	Mean	ah-mean	Angular speed	Mean	w-mean
	Median	ah-median		Median	w-median
	Maximum value	ah-max		Maximum value	w-max
	Minimum value	ah-min		Minimum value	w-min
	Variance	ah-s^2^		Variance	w-s^2^
Target distance	Maximum value	od-max	Relative target speed	Maximum value	rs-max
	Minimum value	od-min		Minimum value	rs-min
Duration of braking		t			

**Table 3:** List of score coefficients.

Parameters	Components						
	F_1_	F_2_	F_3_	F_4_	F_5_	F_6_	F_7_
v-mean	−0.048	0.258	−0.022	0.004	−0.025	−0.013	0.023
v-median	−0.043	0.248	−0.019	−0.002	−0.009	−0.024	0.016
v-max	−0.052	0.222	−0.049	−0.001	0.084	0.054	0.059
v-min	−0.047	0.272	−0.008	0.029	−0.176	−0.073	−0.004
v-s^2^	−0.012	−0.041	−0.102	−0.069	0.322	0.208	0.222
ah-mean	0.206	−0.102	−0.009	0.066	−0.071	0.181	0.040
ah-median	0.199	−0.103	−0.014	0.075	−0.044	0.138	−0.005
ah-max	0.105	−0.053	0.198	0.029	−0.105	0.121	−0.124
ah-min	0.077	−0.022	−0.232	−0.006	0.062	0.022	0.268
ah-s^2^	0.029	−0.015	0.293	−0.030	−0.256	0.078	−0.143
az-mean	−0.043	0.022	0.003	0.438	−0.038	−0.041	−0.029
az-median	−0.024	−0.001	−0.023	0.406	0.033	−0.062	0.075
az-max	−0.045	−0.012	0.223	0.181	0.065	−0.094	0.088
az-min	−0.025	0.046	−0.199	0.150	−0.160	0.037	−0.164
az-s^2^	0.014	−0.036	0.222	−0.036	0.091	−0.086	0.427
w-mean	−0.195	−0.014	0.017	0.073	−0.045	0.106	−0.023
w-median	−0.198	−0.004	0.023	0.073	−0.056	0.099	−0.034
w-max	−0.173	−0.010	0.023	0.049	−0.046	0.379	−0.024
w-min	−0.155	−0.031	−0.004	0.074	−0.021	−0.093	0.000
w-s^2^	0.049	−0.004	−0.014	−0.071	−0.071	0.539	0.055
od-max	−0.034	0.017	−0.007	0.019	0.291	−0.085	−0.065
od-min	−0.003	0.041	−0.014	0.053	−0.006	0.102	0.578
rs-max	0.071	−0.086	−0.046	−0.052	0.379	−0.195	0.070
rs-min	0.085	−0.088	−0.020	−0.085	−0.183	−0.051	0.047
t	−0.017	−0.014	0.039	0.059	0.162	0.049	−0.229

**Table 4:** Braking pattern recognition accuracy of the LDA and LDA2vec models.

Braking Pattern Type	Recognition Accuracy	
	LDA Model	LDA2vec Model
Impulse braking	80.29%	85.23%
Smooth braking	83.98%	86.45%
Gentle braking	81.34%	88.12%

## Data Availability

The data presented in this study are available on request from the corresponding author.
